# Neural Network Model Design for Landscape Ecological Planning Assessment Based on Hierarchical Analysis

**DOI:** 10.1155/2022/1926227

**Published:** 2022-08-29

**Authors:** Jing Liu, Xudan Zhou

**Affiliations:** ^1^School of Xiamen University of Technology, Xiamen, Fujian 361024, China; ^2^College of Forestry and Grassland of Jilin Agriculture University, Changchun, Jilin 130118, China

## Abstract

In this paper, an in-depth study and analysis of landscape ecological planning and evaluation are carried out using the analytic hierarchy process (AHP) algorithm that integrates neural networks. The application of AHP in the field of tree species planning and the introduction of quantitative analysis methods can effectively change the subjectivity of previous qualitative analysis in tree species selection and make it objective, scientific, and reasonable. The research can provide a reference for other urban tree species planning. From the connotation of landscape ecological service process and ecological space structure, the analysis of landscape ecological service process involves service supply area and service association area, which correspond to different key components of ecological space structure. With the help of the platform, based on the identification and identification methods and theories of ecological spatial structure, the key components of ecological spatial structure in different environments are identified and extracted by using the representation model, binary suitability model, weighted suitability model, and process model. The type of service is based on the different service processes supported by the key components of the ecological spatial structure, forming the ecological spatial structure under different service types. Spatial structure; on this basis, the basic characteristics of the key components of the ecological spatial structure are analyzed, and the correlation characteristics of the ecological spatial structure are analyzed based on the correlation classification system of ecological spatial structure. A backpropagation (BP) neural network-based state assessment method of the grid structure is established. The method takes the parameters of the autoregressive model constructed by the acceleration signals of different working conditions as the feature quantity and the results of the fuzzy hierarchical analysis method as the labels, divides the data set into a training set and a test set, and uses the BP neural network learning method and the training set to supervised train the BP neural network learning assessment model. The test set is used to test the effectiveness and accuracy of the BP neural network-based learning method. The study shows that the evaluation system established by the BP neural network structure is fast and accurate and can substantially reduce the cost of manual testing.

## 1. Introduction

With the deepening of theoretical research in ecology and other disciplines, the theoretical and practical methods of landscape ecological planning have been developed in various countries. The early landscape ecological planning was short-sided and relied more on subjective materialistic judgments for design, but with the access and role of these integrated disciplines, the later design increasingly relies on the integrated consideration of multiple disciplines for more reasonable planning and design. In the context of multi-planning, the study of cognitive frameworks provides an opportunity to unify the cognition of more decision-makers, provide methods for clear cognition of sites, and coordinate multiple decision-making to propose suitable planning methods. The practice of landscape ecological planning started late, and the translation of multidisciplinary knowledge into tools to guide space and methods to guide planning practice belong to research gaps. Nowadays, increasingly ecological and environmental problems threaten human life, and the need for new holistic approaches is more urgent [1]. As a disciplinary direction that aims to integrate multiple disciplines to coordinate the relationship between humans and nature, it is necessary to organize the existing landscape cognitive view and research methods to contribute to environmental issues. With the impact of urban construction and public infrastructure in recent years, the surrounding ecological environment has been destroyed, making the ecosystem service function gradually weakened. The hilly and mountainous region of Jiangnan is rich in forest resources, lakes and rivers, and fewer plains, but most of the human socio-economic development is concentrated in the water-rich plains, thus forming a unique landscape unit with complex land use and fragile ecological environment [2].

This paper combines the research methods of cognitive psychology with the practice of landscape ecological planning and composes the historical process of multidisciplinary intervention in landscape ecological planning and design through historical research [3]. People have invested a lot of human and financial resources in the scientific planning of urban wetland parks to achieve the maximum ecological value, but the path of ecological construction of urban wetland parks, rational development, scientific planning, and sustainable development is still being explored. Landscape ecological planning is a scientific planning method that combines the principle of ecological nature with landscape planning, by studying the landscape pattern—the ecological process and the interaction between human activities and the landscape, based on landscape ecological analysis and comprehensive evaluation, the optimal solution and the proposed landscape planning approach, the connotation of which lies in respecting the ecological process and ecological patterns, respecting species diversity, reducing deprivation of resources, maintaining nutrient and water cycles, maintaining the quality of plant habitats and plant and animal habitats, any form of design that minimizes the impact of damage to the environment is called ecological design. To make full use of wetland resources, protect the ecological environment, promote urban-rural integration, and maintain the sustainable development of the city and the environment, landscape ecological planning for urban wetland parks, through the combination of economic planning, environmental planning, and landscape design, so that regional development, resource utilization, and ecological protection are linked to achieving a high degree of unity of economic, social and ecological benefits, and the overall optimization [4]. This is the main way to build a high-quality, harmonious, and livable city and is the way to meet the growing demand of the people for a better life.

The use of hierarchical analysis to plan the garden tree species in Kunming combines quantitative analysis with qualitative analysis, which can achieve scientific and objective tree species planning. The scientific and reasonable planning of urban garden tree species can realize the sustainable development of urban landscaping and make the urban green space system planning effectively play the role of guiding the construction of urban green space and improving the benefits of urban greening. This research result realizes the application of hierarchical analysis to tree species planning, which can verify the rationality of tree species planning in the existing green space system planning and provide a reference basis for the garden tree species planning in other cities. The principles and strategies and methods of landscape planning and design for sponge campus with regional adaptation are proposed, and the proposed strategies and rainwater landscape creation methods are applied to design practice. The paper tries to see the big picture in a small way through this site feature of campus landscape planning and design so that the sponge campus landscape planning and design based on regional adaptation can provide a reference for the planning and design of stormwater landscape in the same type of site in the future.

## 2. Related Work

With the rapid development of the third information technology revolution and the large-scale application of computer technology, the simulation and prediction of stormwater processes using various stormwater simulation software have given rise to the introduction of relevant policies and industry standards for stormwater management [5]. Compared with the traditional stormwater treatment, the new research is more focused on coordinated development and is a forward-looking vision. However, the planning and design of stormwater control in the pavilion have failed to meet expectations in the process of practice because of the lack of relevant laws and regulations to guide and various supporting and coordinating facilities [6]. With the rapid development of the industrial industry in Britain, many laborers moved from the countryside to the cities, and urban housing problems, traffic problems, and environmental problems came to a head. As urban problems became increasingly serious, there was a movement of people moving from big cities to suburban areas to live in idyllic cities, and people took practical actions to pursue a suitable living environment [7]. He argues that high-income residents have a personal stake in urban development and that the gap between rich and poor urban residents can affect the overall livability of cities, so he suggests that the government should reduce the gap between rich and poor urban residents by effectively strengthening control over economic units. Kang has done a lot of research on the theory of livable cities and the human living environment, and he proposes that the construction of livable cities should highlight the concept of being “people-oriented” [8].

The landscape pattern refers to the characteristics and spatial pattern of the landscape component units. Different configurations and combinations of landscape elements form different landscape patterns, and the interaction of different landscape structures and the circulation of different components of material and energy flows in the landscape, i.e., ecological processes, will make the landscape pattern differentiated [9]. General landscape spatial structure characteristics are regular, this law created the landscape pattern of expression. Landscape patterns can reflect the landscape productivity, habitat quality control factors, and ecological stability, according to the pattern of the law of effective prediction of landscape dynamics, in the later stages of the establishment of landscape planning, design, and management objectives [10]. The landscape pattern of wetlands is a special configuration and combination of different landscape elements in the wetland space and is also the result of long interaction between wetland ecological processes and wetland landscape structure [11]. The landscape pattern of artificial wetlands is the principle of human planning and design, respecting the standards of landscape ecological planning, respecting the laws of nature, respecting the normal exchange of materials and energy, and reducing to a minimum all designs that may damage the ecological environment and natural resources to the wetland park planning [12]. The analysis of the landscape pattern can reveal the stability of the ecological environment of urban wetland parks and potential security risks and can also determine the root cause of the generation and control of the landscape pattern [13].

In the studies of small-scale landscape pattern analysis, most scholars only analyze the landscape pattern of a single park, resulting in simple data levels and a lack of comparability, and since there is no standard basis for the meaning of index values, the conclusions of most studies are too subjective and not highly referential, lacking guiding suggestions for park planning and construction in a city, not to mention the geographical environment and urban development conditions different other regions. Using hierarchical analysis to plan garden tree species, combining quantitative analysis with qualitative analysis, can realize the scientific and objective nature of tree species planning. Scientific and reasonable planning of urban garden tree species can realize the sustainable development of urban landscaping and make the urban green space system planning effectively play the role of guiding the construction of urban green space and improving the benefits of urban greening.

## 3. Hierarchical Analysis of Landscape Ecological Planning Assessment Neural Network Model Design

### 3.1. Hierarchical Analysis Fusion Neural Network Model Design

The origin of the artificial neural network is from the neural system in biology class, which is a physical mechanism based on the knowledge of network topology, and simulates the way of human brain processes things by many computing units, and then carries out the mathematical model of distributed transmission. It not only has the ability of self-repeating learning but also imitates the thinking, information processing, and intelligent functions such as storage, recognition, and classification of filtering useless knowledge of the human brain's nervous system to a certain extent claim. The neural network has the following advantages over the human brain system: functions that can be realized by hardware and software together, nonlinear mapping processing capability, non-limitation that can mimic the brain's associative memory, avoidance of complex mathematical models requiring only input and output network topology knowledge, easy distributed parallel computing, fault tolerance and storage capacity, etc.

The structure of artificial neurons is like that of biological neurons, and it is also an abstraction and simulation of the biological nervous system, the so-called abstraction is reflected in the mathematical perspective, and the simulation is reflected in the functional perspective of the structure. From the characteristics and functions of the human brain, a neuron is a signal processing unit. All neurons can have the ability to simulate computer processing information, and their processing steps are divided into three steps: input processing, activation processing, and output processing [14]. Clear awareness of the site, coordination of multi-party decision-making, and proposing appropriate planning methods provide the means. The analysis process takes the product of each input activity and its weight on the neural link, also takes the sum of all weighted inputs to get a total value, and finally passes the transfer function and converts the total value into an output activity. When applying the neural network approach to spatial grid structure state assessment problems, its neuron model often determines the applicable transfer function based on the nonlinear system of the actual spatial grid structure. The information transfer between neurons is also operated by the transfer function.

The hidden layers of a BP neural network generally contain one or more nodes, and there is no linkage between the same layers. The input signal needs to pass through all the nodes in the hidden layer to reach the output node, and the output signal can only affect the output of the nodes in the next layer. As the network processes the data, the neurons are activated and the signals are passed from the input layer to the output layer through the intermediate layers, thus stimulating the neurons in the output layer to produce the input results. The next step corrects the direction of the target output and the actual output by allowing the signal to be passed back from the output layer to the input layer, correcting the connection weights of each layer separately, and this reverse transfer method reduces most of the errors in the input data.

(1) $$\begin{array}{c} z_{k}=f_{1}\left(\sum_{l=0}^{n}v_{ki}^{2}x_{l}^{2}\right),\;k\in Q. \end{array}$$

The output of the output layer node is:

(2) $$\begin{array}{c} y_{i}=f_{2}\left(\sum_{k=0}^{q}w_{ki}^{2}Z_{l}^{2}\right),\;k,i\in M. \end{array}$$

The hierarchical analysis is a decision-making method that decomposes various types of factors affecting the research objectives into different layers, compares the importance of each layer, and translates the comparison results into weight values. It has the advantages of being concise, practical, and easy to operate. In the calculation process of hierarchical analysis, it is necessary to choose suitable weight vector calculation methods, such as the arithmetic mean method, geometric evaluation method, least-squares method, and eigenvector method. The hierarchical analysis is suitable for problems with hierarchical interlaced evaluation indexes and difficult to quantitatively describe the target value of the system [15]. Some scholars often use the hierarchical analysis method in conjunction with the fuzzy integrated evaluation method to study a wider range of problems.

The study of urban livability is a small branch in the field of environmental science, and the establishment of an urban livability index system in Hebei province is a target system problem with interlaced evaluation indexes, and the target values are difficult to be described quantitatively, which is within the scope of application of hierarchical analysis.

According to the judgment matrix established in the previous step, the maximum eigen root max and the corresponding eigenvector W are calculated, and then the consistency test is performed, and if it passes, it is determined as the eigenvector for the next calculation; if it does not pass, the judgment matrix is incorrect due to inconsistency in the judging criteria, and the scaling scoring needs to be performed again to construct a new judgment matrix, and the square root method calculation formula is as follows:

(3) $$\begin{array}{c} M_{i}=\prod_{j=1}^{m}a_{ij}^{m},\;i=1,2,\ldots ,m. \end{array}$$

**Figure 1:**
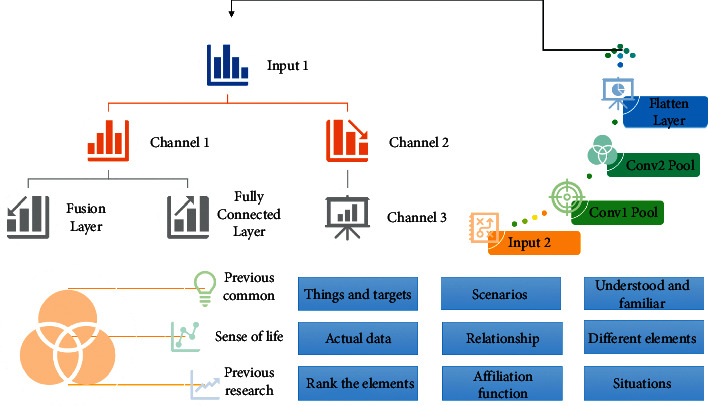
Hierarchical analysis fusion neural network model.

People use the things they know, through the previous common sense of life and previous research to confirm the affiliation function. With a view to contributing to environmental issues. With the impact of urban construction and public infrastructure construction in recent years, the surrounding ecological environment has been destroyed, and the ecosystem service function has been gradually weakened. This strategy is often used for those things and targets that are already well understood and familiar, or for scenarios where it is not easy to get actual data. The strategy is to count the relationship between the importance of different elements in comparison to each other and then rank the elements to determine the affiliation function. This strategy can work better in situations where certain features require many professionals to delineate the boundaries, as shown in Figure 1.

When building an ANN model, the number of nodes in the input layer is related to the number of dimensions one wants to input, and the number of nodes in the output layer is the same as the number of properties in the output, but the number of nodes in the middle layer can be adaptively adjusted according to the actual situation; in the ANN three-layer model shown above, the arrow is the direction of data propagation, and the circle is the neuron node [16]. The calculation process propagated from the bottom input layer to the top output layer, and the weight value of each link is updated through continuous calculation, and the error is back-propagated to make the weight value of each link reach the required value, which finally makes the whole model achieve the optimal prediction effect.

(4) $$\begin{array}{c} \phi =\left(\left\|X_{i}-C_{i}^{2}\right\|\right). \end{array}$$

Radial basis function ANN model: The radial basis function is used as the basis of the hidden layer, and the vector of the input layer of the network is computationally transformed into each neuron node of the hidden layer. When the centroids of each node in the hidden layer are computed using the *k*-means method, then all the computational processes from the input layer to the intermediate layer are fixed. In summary, the process from the input layer to the intermediate layer cannot be expressed using a one-time function, however, the results from the intermediate layer to the output layer are obtained by weighting the results of each hidden layer node and then accumulating all the results, where the weights vary with different data samples or learning times. In this whole process of transformation, it is possible to map the low-dimensional problem to a higher dimension, making the process describable using the network. So, when the output of the hidden layer is known and the expected result, we can answer the weights of each hidden layer neuron by uniting the whole set of linear equations.

By default, a Gaussian function is generally used as the network driving function as shown in equation (5). Its connotation lies in respecting the ecological process and ecological pattern, respecting species diversity, reducing the deprivation of resources, maintaining the nutrient and water cycle, and maintaining the quality of plant habitats and animal and plant habitats.

(5) $$\begin{array}{c} \phi =\exp\left(\left\|X_{i}-C_{i}^{2}\right\|-\frac{e^{2}}{2s^{2}}\right). \end{array}$$

The classification system of ecological spatial structure composition relatedness is the basis for the analysis of ecological spatial structure relatedness characteristics. The classification system of ecological spatial structure composition relatedness is based on landscape ecological service mode and landscape ecological service range and classifies the spatial location relationship among service supply area, service-related area, and service demand area [17]. So far, spatial correlation classification research is relatively sparse, and the cognition of spatial correlation relies heavily on the division of spatial relationship categories between service supply area and service demand area.

**Figure 2:**
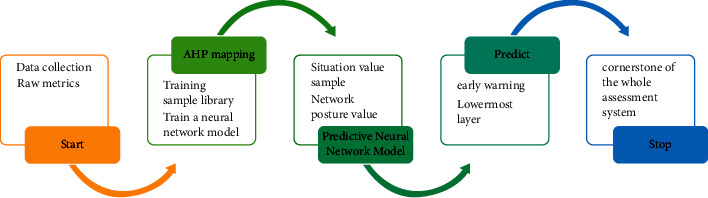
Prediction model.

It is known from the connotation of ecological spatial structure that the landscape ecological service process is the internal correlation mechanism of ecological spatial structure formation, and different types of landscape ecological service processes need different ecological spatial structure characteristic attributes to support. Therefore, to identify the ecological spatial structure, we need to clarify the key components of the ecological spatial structure under different landscape ecological service types to support the ecological service process, and then summarize the key attributes of the ecological spatial structure associated with them, and finally form the key elements to be considered for the identification of ecological spatial structure under various landscape ecological service types to support the corresponding landscape ecological service process, as shown in Figure 2.

The network layer, as the lowermost layer of the model proposed in this paper, is the cornerstone of the whole assessment system and plays a crucial role. The selection of network layer indicators directly determines the reasonableness and accuracy of the whole model in assessing the network operation, so the selection of network layer indicators needs to be carefully combined with various factors. Therefore, this paper first describes how to select the network layer metrics.

Since bandwidth, traffic parameters of the network, and throughput metrics represent similar attributes and are relatively redundant metrics, only one of them needs to be selected to represent the size of the transmitted data volume in the network [18]. Any design form that minimizes the damage to the environment is called designed for ecology. Also, since the false packet rate is not mentioned and not used in previous literature, this paper discards this metric. In summary, this paper selects four metrics, namely, delay, delay jitter, packet loss rate, and throughput, as the metrics of the network layer.

### 3.2. Experimental Design for Landscape Ecological Planning Assessment

The impact of land use and landscape pattern on ecosystem services is mainly expressed in the change of ecosystem service value. This method analyzes the relationship between biotic and abiotic factors in time from a vertical perspective and uses the “lasagna” model to synthesize and filter this knowledge and results to solve the problem. He believes that nature is an evolutionary process, a vertical process in which physical and biological factors are constantly interacting and changing with each other under the action of time, and evolving in concert. It forms a certain value system that can provide certain services and constraints for human life. He suggests combining landscape ecological planning with natural processes and calls on landscape planners to use ecological methods to understand nature: and describe its processes to guide the development and utilization of land.

He sees the landscape as a vertical structure formed by the superposition and interaction of elements and considers the landscape as a vertical unit structure reflecting the intrinsic value of the land. In other words, it is necessary to study in detail the evolutionary laws of natural existence and to have a deep understanding of the characteristics of the landscape to find the appropriate value of the land itself and to realize the most suitable use of the land.

**Figure 3:**
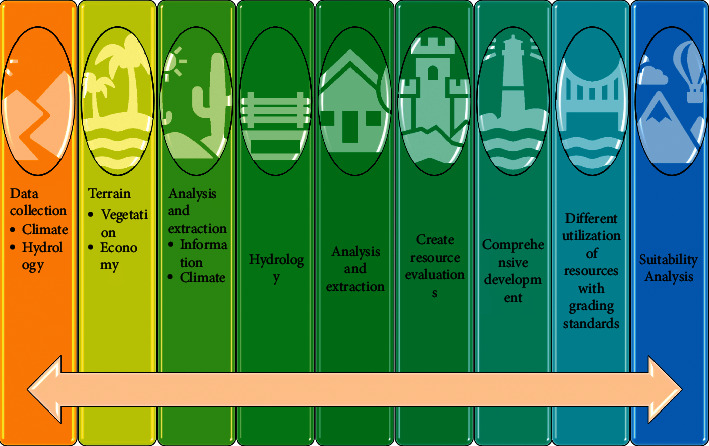
Ecological planning flow chart.

Different habitat units cause different distributions of organisms, and each distribution is the result of mutual matching between organisms and the environment [18]. Organisms are influenced by environmental changes and actively select or adapt to different types of habitat units. The distribution of organisms is best matched to the habitat unit, and the species preserved through adaptation is the result of the best match between ecological factors and the traits of the organisms or the best match between the characteristics and traits of the organisms and the environment in which they live. This match must be made for the organism to survive, as shown in Figure 3.

By investigating and analyzing the natural resources of the area, determining the characteristics of the resources, and judging the ecological performance, analyzing, and evaluating the ability of the land in the area to adapt to development, and thus clarifying the future role and use of the land, a state formed in which the behavioral activities of human modification of nature are consistent and harmonized with the characteristics of the site and natural processes.

The landscape area TA is an index describing the total area of the park, the number of patches NP refers to the number of all patches in the park, and, the density of patches PD refers to the ratio of NP to TA; the ratio of the largest patches to the total area of the park LPI refers to the ratio of the largest patches to the total area of the park and is used to describe the most dominant patches in the park. The size of the area at the landscape level has a great influence on the ecological stability of the whole park, and a park with a large area can play a higher ecological benefit and its ecological stability is also better. The density index, on the other hand, reflects the degree of fragmentation of the landscape, and on the other hand, the density index also reflects the situation of landscape diversity and landscape richness.

**Figure 4:**
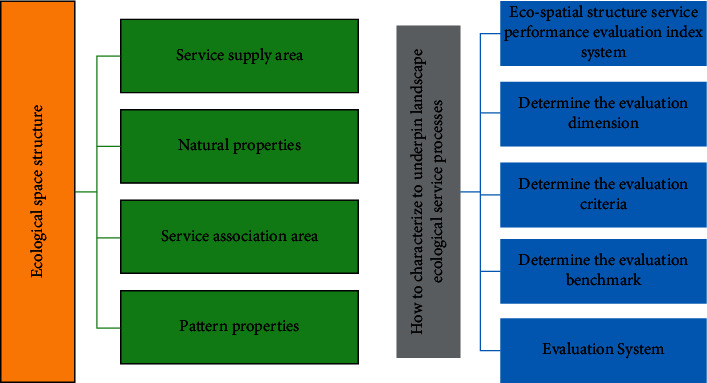
Ecological spatial structure evaluation logic.

Make urban green space system planning effectively play the role of guiding urban green space construction and improving urban greening benefits. The results of this research realize that the analytic hierarchy process is applied to the field of tree species planning, which can verify the rationality of tree species planning in the existing green space system planning. Although the situation faced in different regional scales is complex and varied, because the laws of surface runoff and hydrological cycle are universal, rainwater control methods based on the concept of low-impact development are generally accepted. But at the same time, sponge cities are not equivalent to certain kinds of fixed low-impact development facilities, and specific stormwater site environments should use different solutions and strategies, as shown in Figure 4.

The multi-factor superposition evaluation method is the most basic method among multi-factor comprehensive evaluation methods. This method does not require complex model calculation, directly superimposes each factor affecting the ecological service process of the landscape, uses evaluation criteria to screen and limit the range of key composition areas of ecological spatial structure, and requires high data accuracy because this method directly uses spatial data or relevant map pieces for spatial evaluation analysis. The combination of multiple indicators and data sources is used and the minimum homogeneous geometric area unit is generated by the GIS overlay function, and this method is also the main method for service supply area (SPA) or service demand area (SDA) identification. In addition, regional units are extracted based on quantitative index evaluation results, and most of the index selection involves the depiction of ecosystem conditions and current land use characteristics.

The logical framework of the evaluation of the service performance of the ecological spatial structure shows that the discrimination of the natural and pattern attributes required to support the service supply area (SPA) and service association area (SCA) of different landscape ecological service processes, and the determination of the evaluation benchmark in this way is one of the cores of the research on the evaluation of the service performance of an ecological spatial structure [19–21]. The evaluation of the performance of ecological spatial structure services is based on the natural attributes and pattern attributes. To summarize the service performance evaluation benchmarks of key components of the ecological spatial structure under various landscape ecological service types.

## 4. Results Analysis

### 4.1. Hierarchical Analysis Fusion Neural Network Model Performance Results

In the whole grid structure health state evaluation system, the calculation and rating of the third level performance layer, the second level system layer, and the first level target layer draw on the relevant specifications, and deterministic evaluation. While the third level performance layer indicators are influenced by the fourth level location, stress ratio, and damage degree on the structural state, so combined with this test then the fuzzy comprehensive evaluation method is used to assess the fourth level indicator layer.

**Figure 5:**
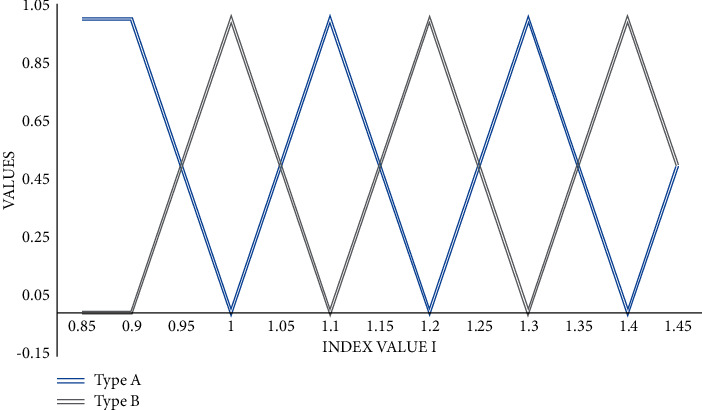
Affiliation function diagram.

The distribution form of the fuzzy problem of a comprehensive evaluation of this test is the same as most of the actual engineering, and it is handled by trapezoidal and approximate triangular distribution, which is combined with common trapezoidal distribution to derive its affiliation function as shown in Figure 5.

The process of the damage state of each component of the whole grid is considered comprehensively in the spatial grid structural health state assessment system, and the influence of location, stress ratio, and damage degree on the structural state is calculated in this paper based on the state assessment method of hierarchical analysis. Then a mathematical model of distributed transfer is performed. It not only has the ability of self-repetitive learning, but also imitates the human brain nervous system's thinking, information processing, storage, identification, and classification of the intelligent functions of filtering useless knowledge to a certain extent and saying.

The least-squares errors for training and testing are shown separately, where the least-squares error for batch size 10 is less than 0.1. The trend of least-squares error throughout training and testing shows that the least-squares error decreases faster in the early stage of training, and increases when the batch size is larger than 10, instead of decreasing the training improvement of the neural network. This is related to the number of layers and the number of units per layer of the selected neural network. To investigate the effect of the number of layers and cells, the number of layers and cell nodes will be adjusted in the subsequent subsections to quantitatively analyze the effect of the number of layers and cells on the data of this experiment.

**Figure 6:**
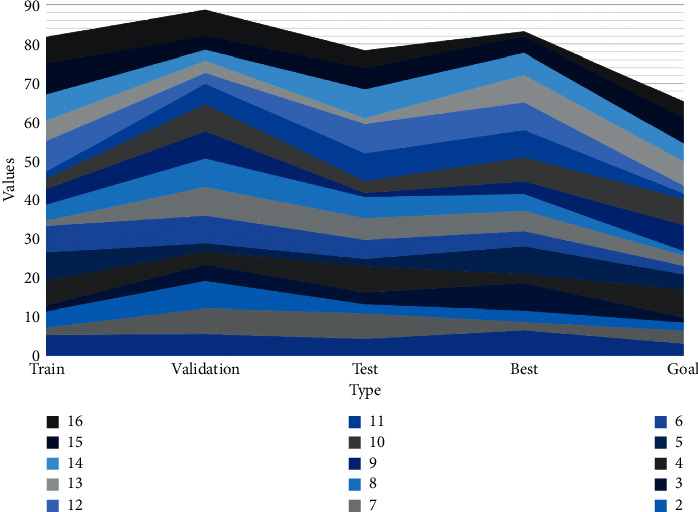
Plot of the inspection of the effect of training via the network.

As shown in Figure 6, the changes in gradient, Mu value, and the error rate of the neural network during the training process are demonstrated. From the gradient graph, it is easy to conclude that the gradient value will slowly decay to 0.010422 as the number of batch layers increases, the gradient decreases continuously as the training progresses, and the gradient is already less than 0.01 when the batch size is 10, which corresponds to the neural network training effect test graph. The Mu value determines whether the model is trained according to the Newton method or the gradient method. As the Mu value increases, the learning process is trained mainly based on gradient descent, and when the iteration makes the error increase, the Mu value increases until the error no longer increases, but if the Mu value is too large, it will cause the learning to stop when the minimum error has been found, which is why the learning is stopped when the Mu value reaches the maximum value. The Mu value represents the weight error tuning. The Mu value represents the area where the range can affect the number of outputs. From Figure 6, the Mu value fluctuates in the first three batch sizes and remains constant at 0.0001 when it is greater than three. When applying the method of the neural network to evaluate the state of the spatial grid structure, the neuron model often determines the applicable transfer function according to the nonlinear system of the actual spatial grid structure. The change in the error rate is consistent with the neural network training effect test plot, where the error rate increases after the batch size of 10.

**Figure 7:**
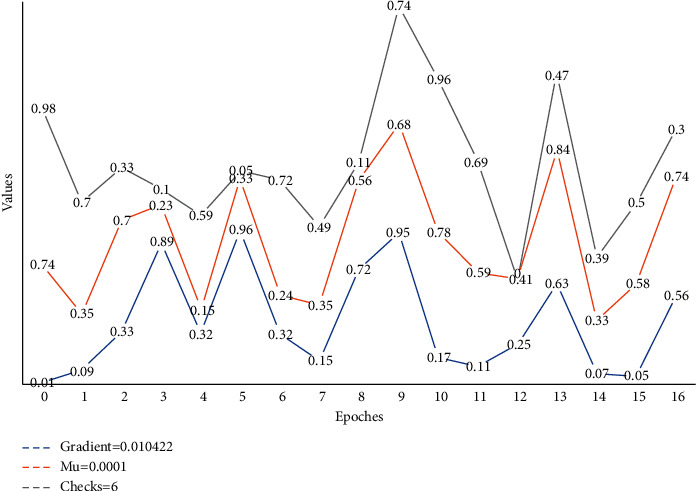
Neural network training state effect graph.

The regression analysis system in the network, as shown in Figure 7, gives a precise linear relationship between the target value and the actual output with *R* = 1. This layout (topology) of the BP neural network is satisfactory.

The overall error of state evaluation using a neural network is relatively uniform, and few local errors very particularly widely, and the number of positive and negative errors is the same, except for some special points where the error is greater than [−1, 1], these special points may be an artificial collision or sudden external noise interference during the test, which are uncontrollable factors, while the rest of the points are within this range. For a more uniform analysis of the error performance, Figure 7 illustrates the percentage distribution of the BP neural network state evaluation error. From the figure, the maximum percentage error is less than 15%, most of the errors are less than 10%, and the errors are evenly distributed. The error plot analysis shows that for the practical application of the BP neural network for state assessment research, engineers, and researchers must consider the existence of assessment errors. Although the neural network-based condition assessment achieves easy and fast identification, before the result is identified, other means including local detection, human observation, etc. must be used to reconfirm the degree of damage and analyze the condition level to prevent the impact of these two types of errors on human life and property.

### 4.2. Experimental Results of Landscape Ecological Planning Assessment

Ecosystem service values showed a weak correlation with patch density (PD), patch average area (AREA-MN), and aggregation (AI), with correlation coefficients of 0.261, 0.442, and 0.665, respectively, at the 0.01 level. The average area of the patches responded to the fragmentation complexity of the landscape pattern, and its fragmentation degree will directly affect the size of the ecosystem service value of the whole study area. The fragmentation of the landscape pattern is related to the land use type, and the fragmentation of woodland and water area is increasing year by year, and the area of woodland and water area is decreasing year by year due to human interference and urbanization, which makes the value of ecosystem services decrease year by year. Therefore, to achieve sustainable development, it should be helpful to protect woodland and watershed ecosystems so that their fragmentation can be reduced and the ecosystem service value of the whole study area can be increased. The value of ecosystem services was not significantly correlated with the correlation coefficient of 0.032 for the walk and juxtaposition index, which describes and calculates the distribution characteristics of ecosystems among various types of blocks. The correlation coefficient indicates that there is no significant correlation between the walk and juxtaposition indices for each tessellation.

**Figure 8:**
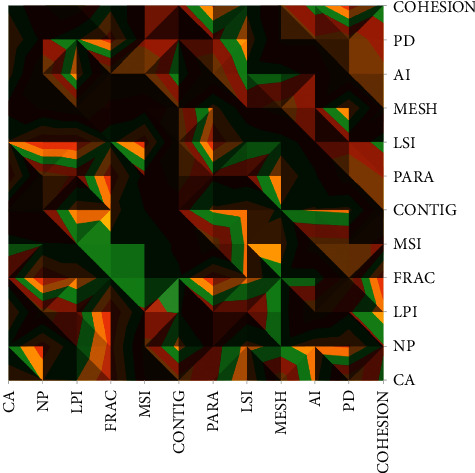
Results of Pearson correlation analysis of landscape type indicators.

There is a strong correlation between ecosystem service values and land use landscape patterns. Changes in land use directly affect changes in landscape patterns and thus indirectly affect changes in ecosystem service values. To improve the value of regional ecosystem services, it is necessary to promote and implement a sustainable economic policy aimed at protecting the environment, realize a social-ecological-economic circular ecological-economic model, and promote the rapid development of the regional economy and society, as shown in Figure 8.

The overall trend of carbon storage in woodlands, water bodies, and built-up areas is increasing. Due to the policy of “two rounds” of returning farmland to forest and the increase of abandonment of rural sloping land, the landscape type has changed more obviously, which has prompted the recovery of woodlands and the advantage of forests in carbon sequestration, resulting in the continuous increase of carbon sequestration in woodlands in the reservoir area. Of course, the increase in carbon sequestration in water bodies does not mean an increase in their carbon sequestration function, but the increase in water bodies in the reservoir area, which floods the original arable land, forest land, and grassland. The built-up area itself has a low carbon storage capacity, and although it has increased to a certain extent during the study period, the expansion of the built-up area inevitably leads to the loss of carbon storage function of other land types from the perspective of the whole study area. In contrast, the total carbon storage in paddy fields, drylands, grasslands, and unused lands tends to decrease. In addition to the decrease in area, the decrease in carbon storage in these three landscape types is also due to the declining carbon sequestration capacity in the conversion of landscape types. It is mainly used in the fields of safety production science and environmental science. Some scholars often combine AHP and fuzzy comprehensive evaluation to study problems in more fields.

**Figure 9:**
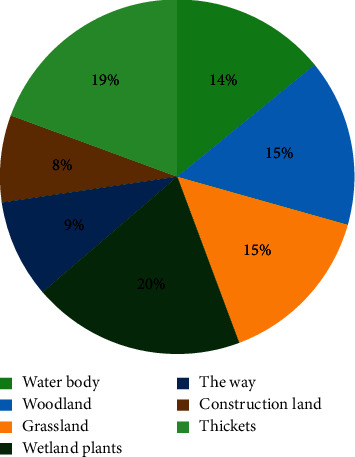
Reference value of patch type area share.

From the current situation of wetland parks, woodland and water bodies are the dominant landscape in wetland parks, and the distribution of water bodies concentrated and woodland is relatively balanced, the distribution of water bodies and woodland is the most obvious; the area of scrub and construction land is generally lower, and their edge effect is also lower, which is caused by their small area or more homogeneous shape. The edges of woodland and roads are generally more complex in wetland parks, and the edges of Belus Bay Wetland Park and the edges of roads in Jincheng Lake Wetland Park are the most complex, as shown in Figure 9.

According to the analysis of the indices of patch type area and density, in addition to roads, the accessibility of its woodland, grassland, and scrub is also higher, and the modularity of roads and construction land is obvious, with roads accounting for 17.5% of the landscape area, which is greater than the average of the three parks' road area ratio of 9%, and construction land accounting for 9.3% of the landscape area ratio, which is higher than the average of the three parks' 4.8% nearly twice, which indicates that its service facilities. This indicates that its service facilities and recreation sites are better and can provide more public service space for the public, but it also increases the degree of landscape fragmentation in the park, with a patch density of 2.5 patches per hectare, which is higher than the average of 1.6 patches per hectare in the three wetland parks. In addition to roads and construction land, the park is not high in terms of area, aggregation, and combination of the remaining landscape types, which are more evenly distributed, and its landscape level has better landscape diversity, higher landscape uniformity, and even distribution of landscape pattern, and richer and more diverse landscape style.

## 5. Conclusion

This paper is guided by the main ideas and theories of garden arboriculture, urban green space system planning, plant landscape planning and design, plant community science, landscape ecology, operations research, and other disciplines. The water-adapted landscape is a traditional ecological heritage that cannot be ignored in building a spongy country and is a spongy landscape with vernacular regional characteristics. The water-adapted landscape, because of its close integration with the natural and human characteristics of the region, has a high regional human connotation and contains the traditional sponge experience and wisdom of adapting to regional hydrological changes. These have important implications for the construction of modern sponge cities, especially when the development site is closely linked to the regional water-adapted landscape, which should be considered in the planning and design of rainwater. The main logic of the framework for evaluating the service performance of the ecological spatial structure is to study whether the ecological spatial structure can efficiently respond to and support the necessary landscape ecological service process, to evaluate the key components of the ecological spatial structure, including the service supply area and the service association area, and to analyze whether the natural and pattern properties of the two parts can effectively support the landscape ecological service process. The service process is the criterion to judge whether the ecological spatial structure can efficiently and sustainably supply the landscape ecological services, and then reflect the service performance level of the ecological spatial structure.

## Data Availability

The data used to support the findings of this study are available from the corresponding author upon request.
